# A retrospective analysis of interobserver variability in 356 cases over a period 10 years: Celiac disease

**DOI:** 10.6026/973206300220137

**Published:** 2026-01-31

**Authors:** Shikha Ghanghoria, Arvind Ghanghoria, Tarun Prakash Verma, Aashita Thakur, Gulladurthi Prudhvi Sai Chownika, Sachin Parmar

**Affiliations:** 1 Department of Pathology, Mahatma Gandhi Memorial Medical College (M.G.M.M.C) and Maharaja Yeshwantrao Hospital (M.Y.H), Indore, Madhya Pradesh, India; 2 Department of General Surgery, Mahatma Gandhi Memorial Medical College (M.G.M.M.C) and Maharaja Yeshwantrao Hospital (M.Y.H), Indore, Madhya Pradesh, India; 3 Multidisciplinary Research Unit, M.G.M Medical College, Indore, Madhya Pradesh, India; 4 Department of Pathology, Mahatma Gandhi Memorial Medical College (M.G.M.M.C) and Maharaja Yeshwantrao Hospital (M.Y.H), Indore, Madhya Pradesh, India; 5 Department Community Medicine, V.K.S. Government Medical College, Neemuch, Madhya Pradesh, India

**Keywords:** Celiac disease, histopathology, marsh-oberhuber, corazza-villanacci, interobserver agreement

## Abstract

Celiac disease (CD) is an immune-mediated enteropathy in which dietary gluten triggers a T-cell-driven inflammatory injury of the small-intestinal mucosa in genetically predisposed individuals. Therefore, it is of interest to compare the distribution of the diagnostic categories and the interobserver variation of the Modified Marsh-Oberhuber and Corazza-Villanacci histopathological categories in biopsy-proven celiac disease samples of 356 cases collected in 10 years. All duodenal biopsies were reviewed by two independent pathologists who scored it twice with each of the two systems; Cohen concordance was used as a measure of interobserver agreement. Majority of initial diagnoses were compressed in Marsh III subtypes and recategorization showed significant discordance of Marsh (iii) a to (iii) c, which represents little reproducibility. By contrast, Corazza-Villanacci produced majority of Grade B1 tasks with maximal consensus among pathologists, which was highly reproducible and did not compromise clinically valuable stratification. The results argue in favor of the straightforward implementation of the streamlined Corazza-Villanacci system in Indian high throughput settings to increase the consistency of the diagnoses and standardize patient care.

## Background:

Celiac disease (CD) is a T-cell-mediated intestinal epithelial disorder triggered by gluten in the diets of predisposed people, with a worldwide incidence of about 1 percent. A long clinical manifestation spectrum that covers asymptomatic and extremely malabsorbed features. The gold-standard is the histological examination of the small intestine, which can directly diagnose such clinical parameters as the architecture of the villi, hyperplasia of crypts and increased intraepithelial lymphocytosis. The original Marsh classification described a continuum of mucosal abnormalities ranging between normal villi and villous atrophy with elevated intraepithelial lymphocytes (IELS), through to complete villous atrophy including morphometric measurements that were not practical to use routinely in diagnosis [1]. The work of Oberhuber and colleagues refined Type III threefold (Type (iii) a: partial atrophy; Type (iii) b: subtotal atrophy and Type (iii) c: atrophy) resulting in the six-level Marsh-Oberhuber system used in clinical practice [2]. The resulting higher granularity, however, brought in the possibility of subjectiveness and complexity by creating issues regarding the inability to replicate the results to different observers. In response to these shortcomings, Corazza and Villanacci in 2005 suggested downsizing the two (Grade A (non-atrophic, IEL count >=25 per 100 enterocytes) and Grade B (atrophic) with B1 (partially atrophic) and B2 (totally atrophic) [3]. Such categorization is dichotomous and is supposed to minimize the complexity of the classifications and maintain the important clinical distinctions. Various researchers have determined interobserver agreement through Cohen kappa since its development, with the latter interpreted under the scale of agreement developed by Landis and Koch: poor (0.20), fair (0.21-0.40), moderate (0.41-0.60), good (0.61-0.80) and very good (0.81-1.00) [4]. In earlier multicenter studies that assessed the reproducibility of the Marsh Oberhuberversus Corazza Villanacci systems, a mean kappa value of 0.35 (fair) and 0.55 (moderate), respectively were observed to be better with the simplified classification [5]. This study adds practical guidance for high-volume settings by linking "most reliable classification" to consistency between pathologists, which can improve standardization of reporting and downstream clinical decision-making. Therefore, it is of interest to determine interobserver heterogeneity in the histologic grading of CD using the Marsh- Oberhuber histological grading and that of Corazza-Villanacci using kappa value in evaluating which method is the most reliable to be used in routine and research fields.

## Materials and Methods:

This retrospective observational study was conducted on 356 patients diagnosed with celiac disease over a 10-year period, based on duodenal biopsy findings classified initially according to the Modified Marsh-Oberhuber classification.

## Histopathological review:

Archived biopsy slides and reports were retrieved from the pathology department records. All specimens were reassessed independently by two experienced gastrointestinal pathologists, who were blinded to each other's evaluations and to all clinical and serological details. Each pathologist reviewed every specimen twice, classifying them first using the Modified Marsh-Oberhuber system and then according to the Corazza-Villanacci classification. The initial histopathological grades (Marsh-Oberhuber) recorded at the time of diagnosis were also documented for comparison.

## Classification systems:

[1] Modified Marsh-Oberhuber classification: Categorizes duodenal mucosa into six stages (Marsh 0-IV), describing changes from normal architecture with increased intraepithelial lymphocytes (IELS) to complete villous atrophy, with subdivision of Marsh III into partial (iii) a, subtotal (iii) b and total villous atrophy (iii) c.

[2] Corazza-Villanacci classification: Simplifies grading into non-atrophic (Grade A)- normal villous architecture with increased IELS - and atrophic types, which include Grade B1 (partial villous atrophy) and Grade B2 (complete villous atrophy).

## Statistical analysis:

Interobserver agreement between the two pathologists for both classification systems was assessed using Cohen's kappa (κ) statistic in SPSS version 20.0 (IBM Corp., Armonk, NY, USA).

The strength of agreement was interpreted according to the revised Landis and Koch scale by Altman as follows:

[1] Poor: ≤0.20

[2] Fair: 0.21-0.40

[3] Moderate: 0.41-0.60

[4] Good: 0.61-0.80

[5] Very good: 0.81-1.00

## Results:

Table 1 shows Modified Marsh-Oberhuber Classification of Celiac Disease Histopathology that described different grades depending on the amount of intraepithelial lymphocytes (IELS), crypt hyperplasia, or villous atrophy as observed in jejunum and duodenum. They go through grades in the pathological stages of a normal tissue (Marsh 0) through a complete atrophy (Marsh 4) with the significance of severity being expressed through the higher Marsh grades. Table 2 shows the distribution of cases of 356 cases under the classification of Marsh-Oberhuber. Most of the cases are of the Marsh III subvarieties with different levels of villous atrophy. Only a little percentage falls within Marsh 0 or Marsh I/II which depicts mild or normal changes. Table 3 shows that the reclassification of the same 356 cases by two pathologists was inconsistent and it is especially true in the Marsh III subtypes. This draws emphasis on the inter observer variability of this detailed grading system where there are any disparities when it comes to allotment of grades. The numbers of diagnoses based on the Corazza-Villanacci classification of 356 cases in initial diagnosis are observed in Table 4. A majority of them are classified as Grade B1, which implies a partial villous atrophy and the crypt hyperplasia. Fewer patients fall into the Grade A category (normal villi with elevated IELS) or the Grade B2 (villous atrophy of total villous). It is depicted in Table 5 that two pathologists have reclassified the cases depending on the Corazza-Villanacci system. The findings are reproducible amongst pathologists, of which the most common will be Grade B1 representing partial villous atrophy. This interrater reliability supports the ease of the Corazza system as opposed to the Marsh classification. Table 6 is a comparison of the distribution of cases by the Marsh-Oberhuber and Corazza-Villanacci systems. It depicts the concourse of initial and re-classified data of each system by two pathologists. It is important to note that not all grades can be mapped directly onto another; nevertheless, the table intends to show similarities and differences in the classification of cases.

## Discussion:

This study compared the diagnostic distributions of two systems of grading celiac disease by histopathologic criteria and their agreement between and among observers Modified Marsh-Oberhuber and Corazza-Villanacci categories in the cohort of 356 biopsy-proven cases. The noted higher interobserver agreement with Corazza-Villanacci system has been recorded in several prior studies in investigating the same dilemma. Default work by Corazza *et al.* [4] established an average value of kappa of 0.35 (fair) of the Marsh-oberhuber classification and 0.55 of the new Corazza system in a sample of 60 patients using six pathologists. In the same way, Douida *et al.* [6] found mean kappa of 0.48 (moderate) in Marsh-Oberhuber and 0.61 (good) in Corazza of 69 cases evaluated by two pathologists. The findings have been replicated irrespective of a wide geographical variations with Ghanghoria *et al.* [7] indicating a higher level of agreement in the Corazza-Villanacci system (kappa 0.98) over the Oberhuber classification (kappa 0.77) in a retrospective study involving 200 cases. The problem of interobserver variance in histopathology of celiac disease goes right past choice of classification system to the basic question of consistency of diagnosis. Denholm *et al.* [8] carried out a large digital pathology study that looked at 100 whole-slide images and observed a mean previously unseen, coming out to be only 0.73 and kappa coefficients of 0.59. This approximate consensus raised to a higher degree of 0.80 probability and 0.67 kappa when incorporating serological data, thus, attesting to the relevance of clinical circumstances to the evaluation of histopathology. A study of prevented that examined biopsies that had undergone a multinational cohort of 98 cases also had a satisfactorily reported agreement in the detection of villous atrophy (κ = 0.84) but much less corroboration when evaluating the severity of lesions as Corazza-Villanacci classification (κ = 0.39) was significantly higher in concordance than the Marsh- Oberhuber system (κ = 0.31) [9]. Reproducibility issues are the possible solutions with the emerging quantitative methodologies. A study of 486 subjects (261 in derivation cohort and 225 in validation cohort) by Das *et al.* [10] used computer-assisted analysis of digital histological images to develop a quantitative histological classification system showing greater interobserver (24.6 - 71.5%) and intra-observer (69.7 -85.0%) agreement than all the available qualitative classification systems. They applied objective thresholds such as the counts of intraepithelial lymphocytes 25/100 epithelial cells, adjusted fold change in villous height 0.7 and crypt depth-to-villous height ratio 0.5 that have a sensitivity of 90.3% and the specificity is 93.5 to arrive at the diagnosis of celiac disease. Continuous villous height to the crypt depth ratios and intraepithelial lymphocyte densities found through such quantitative morphometric methods have been successful in clinical trials where linear dose-response relationships can be found through gluten challenge studies [11]. The emergence of artificial intelligence and machine learning into the world of pathology is the frontier in solving the problem of variability in diagnosis. Reviewing the current literature, a fantastic step has been achieved, as even ML models could attain the diagnostic capabilities of pathologists when it comes to the detection of celiac disease. An AI tool developed in Cambridge community and trained on 3383 whole-slide-images showed an accuracy of 97 percent diagnostic accuracy, sensitivity above 95 percent and specificity above 95 percent [12]. It is noted that concordance studies have shown that pathologists were equally likely to agree with the AI rather than with other human pathologists and thus it was shown that machine learning can perform as well as humans on a diagnosis, as well as possibly reducing subjective variability [12, 13]. Such digital pathology methods, however, do have the added benefit of quantitative and continuous results compared to categorical results that may not reflect subtle morphological variation which may be missed with more familiar grading systems. Academic interest can only go so far in terms of clinical implications in terms of improved diagnostic reproducibility, but the important effects are on the actual patient. The uncertainty and delay in developing a treatment plan and variability in the assessment of the therapeutic effect might be a result of variability in the histopathological diagnosis. These issues are corroborated by the simplified system by Corazza-Villanacci who in his simplified system provided a framework that addresses these issues in that the system is clinically relevant and more consistent in the diagnosis. This becomes especially significant in the age of the development of the therapeutics of celiac disease, when the regulatory bodies would mandate valid histological endpoints to be used in clinical trials [12, 14]. The three-tier stratification of the system differentiates normal mucosa with increased IEL but not to the level of villous atrophy, rare, adherent, or patchy, making it more granular enough to support clinical decision-making along the lines of the subtypes in the Marsh III without the subjectivity that renders it weak.

## Conclusion:

Corazza-Villanacci classification provided higher interobserver agreement and is more practical for routine use in resource-limited Indian settings.Grade B1B1 best described the most common finding in our cohort (partial villous atrophy), avoiding subjective Marsh III subtyping. Its adoption may improve diagnostic consistency and standardize patient management.

## Figures and Tables

**Table 1:** Modified marsh-oberhuber classification of celiac disease histopathology

**Marsh Type**	**IEL/100 Enterocytes: Jejunum**	**IEL/100 Enterocytes: Duodenum**	**Crypt Hyperplasia**	**Villous**
0	< 40	< 30	Normal	Normal
1	> 40	> 30	Normal	Normal
2	> 40	> 30	Increased	Normal
3a	> 40	> 30	Increased	Mild Atrophy
3b	> 40	> 30	Increased	Marked Atrophy
3c	> 40	> 30	Increased	Complete Atrophy
4	> 40	> 30	Atrophic	Flat

**Table 2:** Initial diagnosis (marsh-oberhuber classification) for 356 cases

**Marsh-Oberhuber Grade**	**Number of Cases**
Marsh 0 (Normal)	15
Marsh I	22
Marsh II	10
Marsh IIIa	110
Marsh IIIb	90
Marsh IIIc	99
Marsh IV	10
Total	356

**Table 3:** Reclassification (Marsh-Oberhuber Classification) by Pathologists

**Marsh-Oberhuber Grade**	**Pathologist 1**	**Pathologist 2**
Marsh 0 (Normal)	14	13
Marsh I	18	20
Marsh II	8	6
Marsh IIIa	128	101
Marsh IIIb	80	100
Marsh IIIc	101	107
Marsh IV	7	9
Total	356	356

**Table 4:** Initial Diagnosis (Corazza-Villanacci Classification) for 356 Cases

**Corazza Grade**	**Interpretation**	**Number of Cases**
Grade A	↑IELS, normal villi	38
Grade B1	Villous atrophy, crypt hyperplasia - partial	185
Grade B2	Total villous atrophy	133
Total		356

**Table 5:** Corazza-Villanacci Classification

**Corazza Grade**	**Interpretation**	**Pathologist 1**	**Pathologist 2**
Grade A	↑IELS, normal villi	41	38
Grade B1	Villous atrophy, crypt hyperplasia - partial	183	182
Grade B2	Total villous atrophy	132	136
Total		356	356

**Table 6:** Distribution of cases by grade - all systems

**Grade**	**Initial Diagnosis (Marsh-Oberhuber)**	**Pathologist 1 (Marsh-Oberhuber)**	**Pathologist 2 (Marsh-Oberhuber)**	**Initial Diagnosis (Corazza-Villanacci Classification)**	**Pathologist 1 (Corazza)**	**Pathologist 2 (Corazza)**
Type 0	15	14	13		-	-
Grade A/ Type Marsh I	22	18	20	38	41	38
Marsh II	10	8	6		-	-
Grade B1/Marsh iiia	110	128	101	185	183	182
Type Marsh iiib	90	80	100	-	-	-
Grade B2/Type Marsh iiic	99	101	107	133	132	136
Type Marsh IV	10	7	9	-	-	-
